# DKC1 aggravates gastric cancer cell migration and invasion through up-regulating the expression of TNFAIP6

**DOI:** 10.1007/s10142-024-01313-2

**Published:** 2024-02-20

**Authors:** Huihua Chen, Yibo Wu, Yancheng Jiang, Zixuan Chen, Tingjin Zheng

**Affiliations:** Department of Clinical Laboratory, Quanzhou First Hospital Affiliated to Fujian Medical University, No. 248 East Street, Quanzhou, 362000 Fujian China

**Keywords:** DKC1, TNFAIP6, Gastric cancer, Migration, And Invasion

## Abstract

Gastric cancer (GC) is one hackneyed malignancy tumor accompanied by high death rate. DKC1 has been discovered to serve as a facilitator in several cancers. Additionally, it was discovered from one study that DKC1 displayed higher expression in GC tissues than in the normal tissues. Nevertheless, its role and regulatory mechanism in GC is yet to be illustrated. In this study, it was proved that DKC1 expression was upregulated in GC tissues through GEPIA and UALCAN databases. Moreover, we discovered that DKC1 exhibited higher expression in GC cells. Functional experiments testified that DKC1 accelerated cell proliferation, migration, and invasion in GC. Further investigation disclosed that the weakened cell proliferation, migration, and invasion stimulated by DKC1 knockdown can be reversed after TNFAIP6 overexpression. Lastly, through in vivo experiments, it was demonstrated that DKC1 strengthened tumor growth. In conclusion, our work uncovered that DKC1 aggravated GC cell migration and invasion through upregulating the expression of TNFAIP6. This discovery might highlight the function of DKC1 in GC treatment.

## Introduction

Gastric cancer (GC) is one of the most usual pernicious tumors of the digestive tract (Correa 2013; Crew and Neugut 2006). The death rate of GC ranks second among all pernicious tumors (Ang and Fock 2014). At present, the detection rate of early GC in China is low. Lymph node metastasis or even distant organ metastasis has been found in most GC patients at first diagnosis, which could not be cured through surgery and affected the prognosis and survival rate of patients (Karimi et al. 2014; Tan 2019; Na et al. 2021). Despite the continuous development of endoscopic technology and the remarkable progress of immunotherapy, the current GC therapeutic effect is restricted, so the death rate of GC in China keeps high (Zong et al. 2016; Zhang and Yu 2020; Sezik et al. 2021; Sun et al. 2021). Therefore, distinguishing novel biomolecules and signaling pathways may supply potential therapeutic strategies for GC.

The dyskeratosis congenita 1 (DKC1) gene was first determined in dyskeratosis congenita (DC) (Dokal 2011; Hisata et al. 2013). Recent reports suggested that DKC1 expression is dysregulated in diversified human cancers, affecting tumor growth or metastasis. For instance, DKC1 facilitates HIF-1α transcription to strengthen angiogenesis and metastasis in colorectal cancer (Hou et al. 2020). In lung adenocarcinoma, suppression of DKC1 stimulates telomere-associated senescence and apoptosis (Kan et al. 2021). In addition, DKC1 exhibited higher expression in glioma and accelerate tumorigenesis and tumor metastasis (Miao et al. 2019). DKC1 stimulates the NF-κB pathway to aggravate cell migration and invasion in clear cell renal cell carcinoma (Zhang et al. 2018). Moreover, lncRNA MEG3 interacts with DKC1 to retard non-small cell lung cancer progression (Yang et al. 2020). Importantly, one study revealed that the positive expression of DKC1 in GC tissues was dramatically higher than that in normal tissues (Kim et al. 2012), but the function and regulatory mechanism of DKC1 in GC were indistinct, so it was investigated.

In our study, the purpose is to probe the regulatory effects of DKC1 on the malignant behaviors of GC. Our results disclosed that DKC1 exhibited higher expression, and DKC1 upregulated TNFAIP6 expression to aggravate the tumor growth and metastasis in GC. This discovery may offer a novel bio-target for GC targeted therapy, mitigating the tribulation of GC patients.

## Materials and methods

### Cell lines and cell culture

The GC cell lines (AGS, HGC27, MKN45, and SNU-1) and gastric epithelial cell line (GES-1) were adopted from the American Type Culture Collection (ATCC; Manassas, VA, USA). The Roswell Park Memorial Institute-1640 (RPMI-1640) medium with 10% fetal bovine serum (FBS), penicillin, and streptomycin was added to culture these above cells in a moist incubator with 5% CO_2_ at 37 °C.

### Transfection

The small interfering RNA (siRNA) against DKC1 (si-DKC1#1, si-DKC1#2), si-negative control (si-NC), pcDNA3.1 target TNFAIP6 (OE-TNFAIP6), and pcDNA3.1-NC (vector) were acquired from Genepharma (Shanghai, China). Next, the transfection of these above plasmids into MKN45 and AGS cells was conducted through Lipofectamine 3000 (Invitrogen).

### Western blot

The lysis of GC cells was done through RIPA lysis buffer (Beyotime, Shanghai, China). Then, sodium dodecyl sulfate–polyacrylamide gel electrophoresis (SDS-PAGE, 10%) was utilized to separate these isolated proteins, followed by moving them to polyvinylidene fluoride (PVDF) membranes (Amersham, USA). Being sealed with non-fat milk, primary antibodies including DKC1 (ab93777, 1/2000, Abcam), HIF-1α (ab51608, 1/1000, Abcam), TNFAIP6 (ab204049, 0.1 µg/mL, Abcam), and β-actin (ab8226, 1 µg/mL, Abcam) were mixed with the membranes for incubation overnight at 4 °C. Next, the secondary antibody (ab6721, 1/2000, Abcam) was added into the membranes for 2 h. After rinsing, the visualization of protein blots was performed through the ECL chemiluminescent detection system (Thermo Fisher Scientific).

### CCK-8 assay

GC cells were grown on the 96-well plate. The addition of CCK-8 solution (Beyotime, Beijing, China) was performed at 0, 24, 48, and 72 h. After 4 h incubation, the OD value (450 nm) was measured through a microplate reader (Bio-Rad Laboratories, Hercules, CA).

### EdU assay

Briefly, GC cells were subjected to EdU (50 μm, RiboBio, Guangzhou, China) for 2-h incubation. Afterward, the GC cells were stained through Apolo and 4′,6-diamidi-diamidino-2-phenylindole (DAPI). Lastly, a fluorescence microscopy was utilized for assessing EdU-positive cells.

### Transwell assay (for invasion)

Transwell chambers (pore size, 8 μM; Corning, NY, USA) pre-coated with Matrigel (BD Biosciences, Franklin Lakes, NJ, USA) were employed for examining cell invasion. The serum-free medium (200 μL) and GC cells were added into the upper chamber, and the medium with 20% FBS (600 μL) was supplemented into the lower chamber. After 48 h, invaded cells were subject to the fixation with 4% paraformaldehyde and staining with 0.1% crystal violet. Ultimately, the invaded cells were evaluated through a microscope (Leica, Wetzlar, Germany).

### Wound healing assay

GC cells were labeled at the six-well plate until 90% confluence. These cells were scratched with a 100-μL sterile tip. At last, at 0 h and 24 h, an Olympus optical microscope was adopted to observe the linear changes.

### In vivo assay

The animal experiments were conducted with the approval of the Ethics Committee of Quanzhou First Hospital Affiliated to Fujian Medical University. The 6-week-old male BALB/c nude mice procured from Charles River (Beijing, China) were separated into two groups (*n* = 5 for each group): the sh-NC and sh-DKC1#1 group. Transfected AGS cells were subcutaneously inoculated into the right flank of mice. Every 7 days, the tumor volume was evaluated. After 35 days, the mice were sacrificed, and the weight of tumors was estimated.

### IHC assay

The paraffin-embedded tissue sections (4 μm) were subject to dewaxing and rehydration. After sealing, the sections were cultured with DKC1, HIF-1α, TNFAIP6, and ki-67 antibodies at 4 °C overnight, followed by mixing with secondary antibody (1:500, ab6112, Abcam, Shanghai, China). Next, the dyeing with diaminobenzidine (DAB) and re-dyeing with hematoxylin were carried out. Images were gained through a microscope (Nikon, Tokyo, Japan).

### Statistical analysis

SPSS version 20.0 software (SPSS, Chicago, USA) was employed for statistical analysis. Data were displayed as the mean ± standard deviation (SD). All experiments were done three times. Difference estimation in two or multiple groups was carried out through the Student’s *t* test or one-way ANOVA. The *p* < 0.05 was set as statistically significant.

## Results

### DKC1 exhibited higher expression in GC

**Fig. 1:**
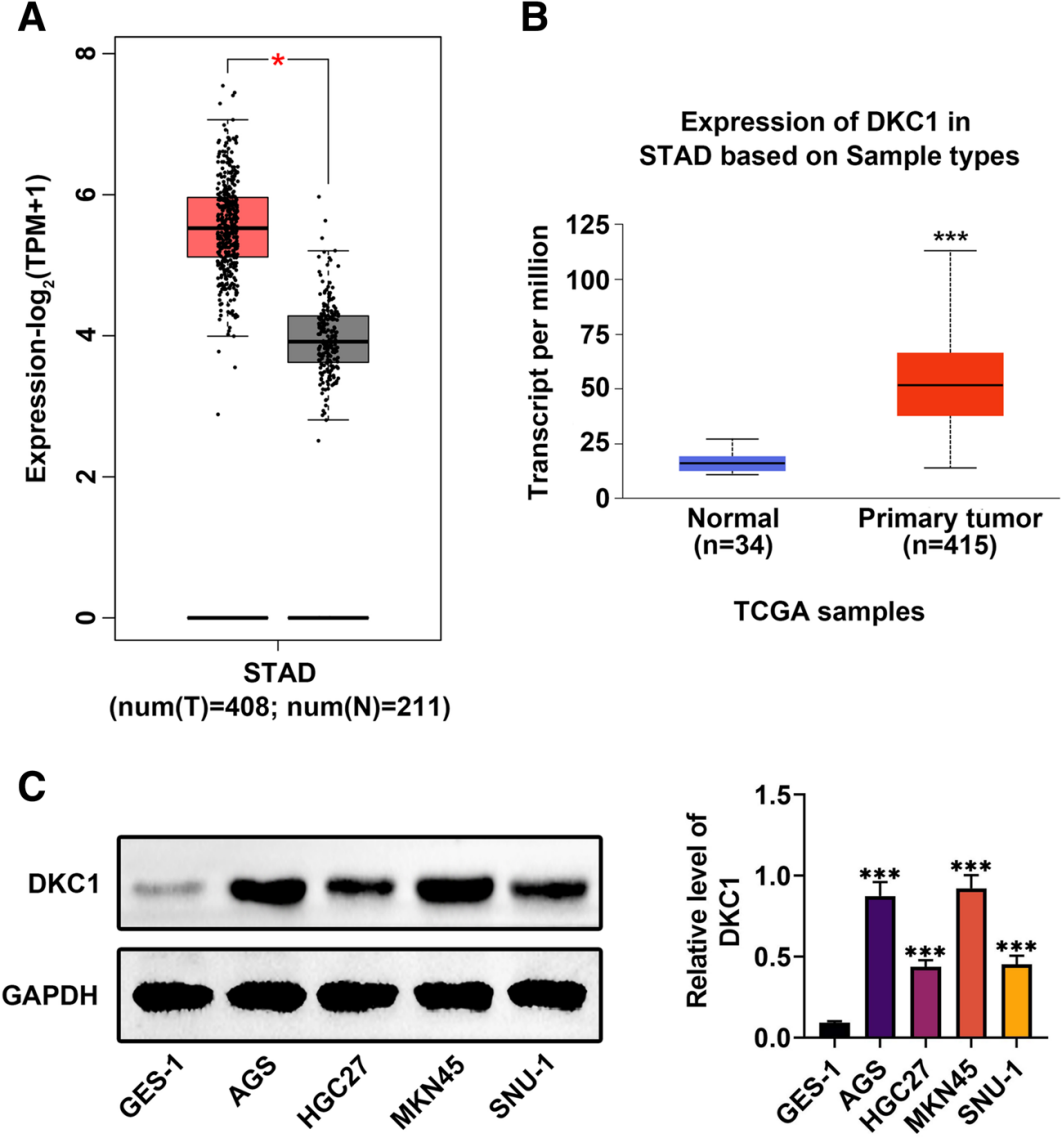
DKC1 exhibited higher expression in GC. **A** The DKC1 expression in STAD tissues was confirmed through GEPIA online database (gray: normal tissues; red: tumor tissues). **B** The DKC1 expression in STAD tissues was confirmed through UALCAN online database (Blue: Normal tissues; Red: Tumor tissues). **C** The protein expression of DKC1 was examined in gastric epithelial cell line (GES-1) and GC cell lines (AGS, HGC27, MKN45 and SNU-1) through western blot. *N* = 3. *p < 0.05, ***p < 0.001

At first, through GEPIA (Gene Expression Profiling Interactive Analysis) and UALCAN (A Portal for Facilitating Tumor Subgroup Gene Expression and Survival Analyses) databases, it was discovered that DKC1 was upregulated in stomach adenocarcinoma (STAD) tissues in comparison with the normal tissues (Fig. 1A, B) (*p* < 0.05). In addition, the protein expression of DKC1 was higher in GC cell lines (AGS, HGC27, MKN45, and SNU-1) than in gastric epithelial cell line (GES-1) (Fig. 1C) (*p* < 0.001). These data revealed that DKC1 exhibited higher expression in GC.

### DKC1 accelerated cell proliferation in GC

**Fig. 2:**
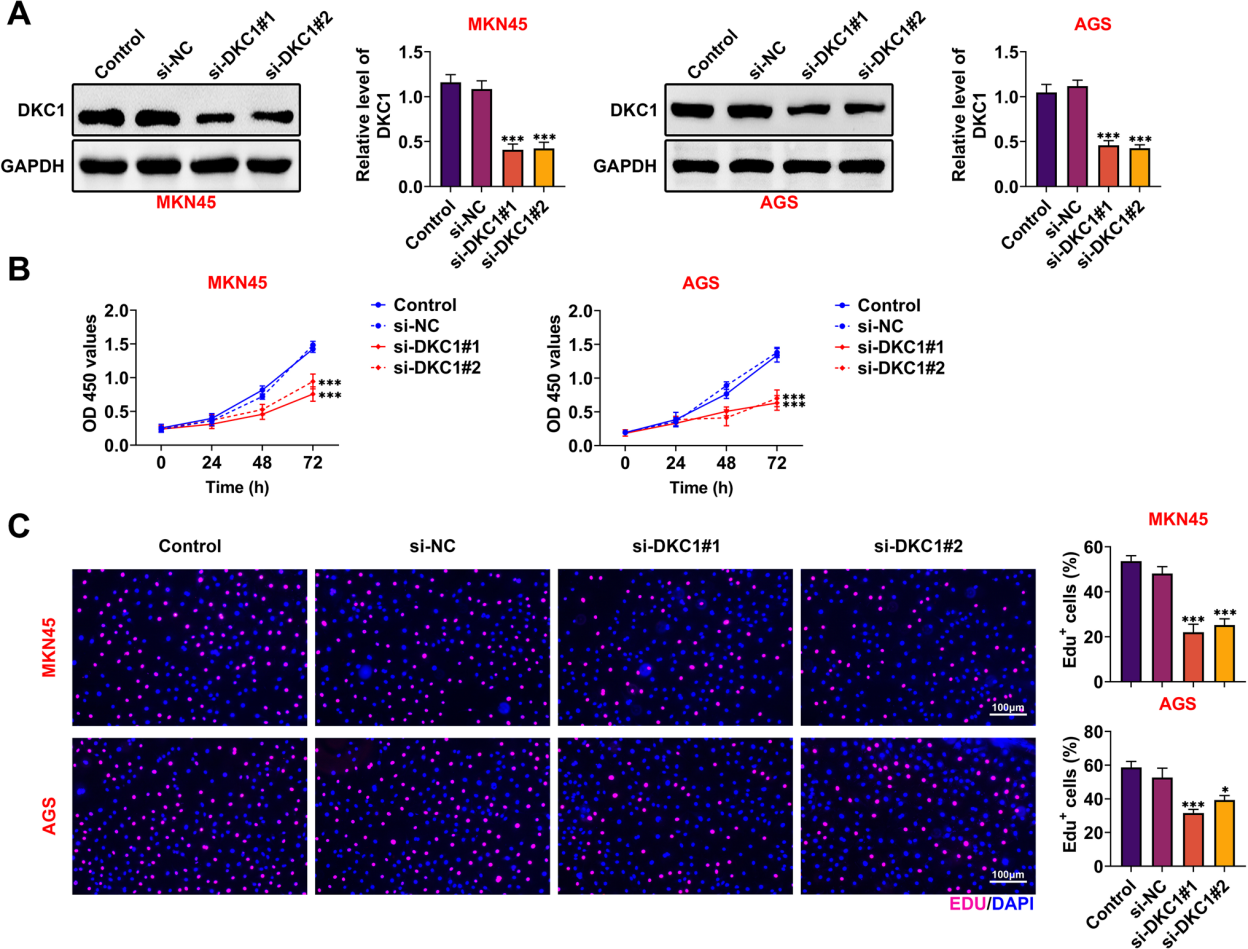
DKC1 accelerated cell proliferation in GC. **A** The knockdown efficiency of DKC1 was identified in MKN45 and AGS cells through western blot. **B**, **C** The cell proliferation was measured after silencing DKC1 in MKN45 and AGS cells through CCK-8 and Edu assay. *N* = 3. *p < 0.05, ***p < 0.001

DKC1 has been affirmed to be a serviceable facilitator in diversiform cancers (Hou et al. 2020; Kan et al. 2021; Miao et al. 2019; Zhang et al. 2018; Yang et al. 2020; Kim et al. 2012), but its function in GC maintains dimness. Therefore, more functional experiments for DKC1 were done for further investigations. As manifested in Fig. 2A, the knockdown efficiency of DKC1 was confirmed (*p* < 0.001). The cell viabilities of MKN45 and AGS cells were diminished after silencing DKC1 (Fig. 2B) (*p* < 0.001). Moreover, it was demonstrated that the EdU-positive cells were decreased after DKC1 suppression (Fig. 2C) (*p* < 0.05). Taken together, DKC1 accelerated cell proliferation in GC.

### DKC1 facilitated cell migration and invasion in GC

**Fig. 3:**
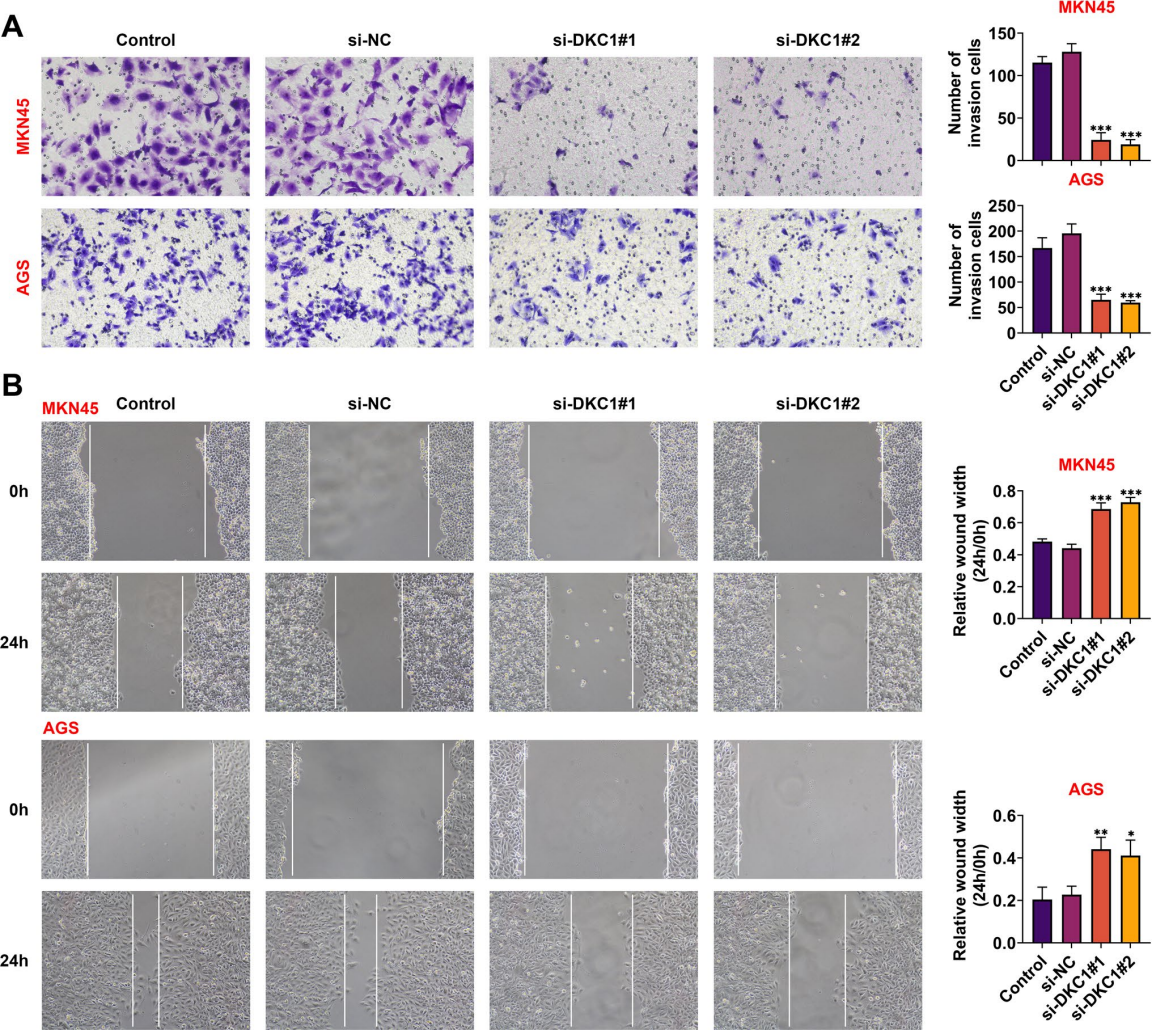
DKC1 facilitated cell migration and invasion in GC. **A** The cell invasion was tested after suppressing DKC1 in MKN45 and AGS cells through Transwell assay. **B** The cell migration was detected after DKC1 knockdown in MKN45 and AGS cells through wound healing assay. *N* = 3. *p < 0.05; **p < 0.01; ***p < 0.001

The invasion abilities of MKN45 and AGS cells were weakened after inhibiting DKC1 (Fig. 3A) (*p* < 0.001). In addition, through wound healing assay, it was indicated that DKC1 suppression markedly reduced the migration ability of GC cells (Fig. 3B) (*p* < 0.05). These results identified that DKC1 facilitated cell migration and invasion in GC.

### DKC1 modulated TNFAIP6 to aggravate GC progression

**Fig. 4:**
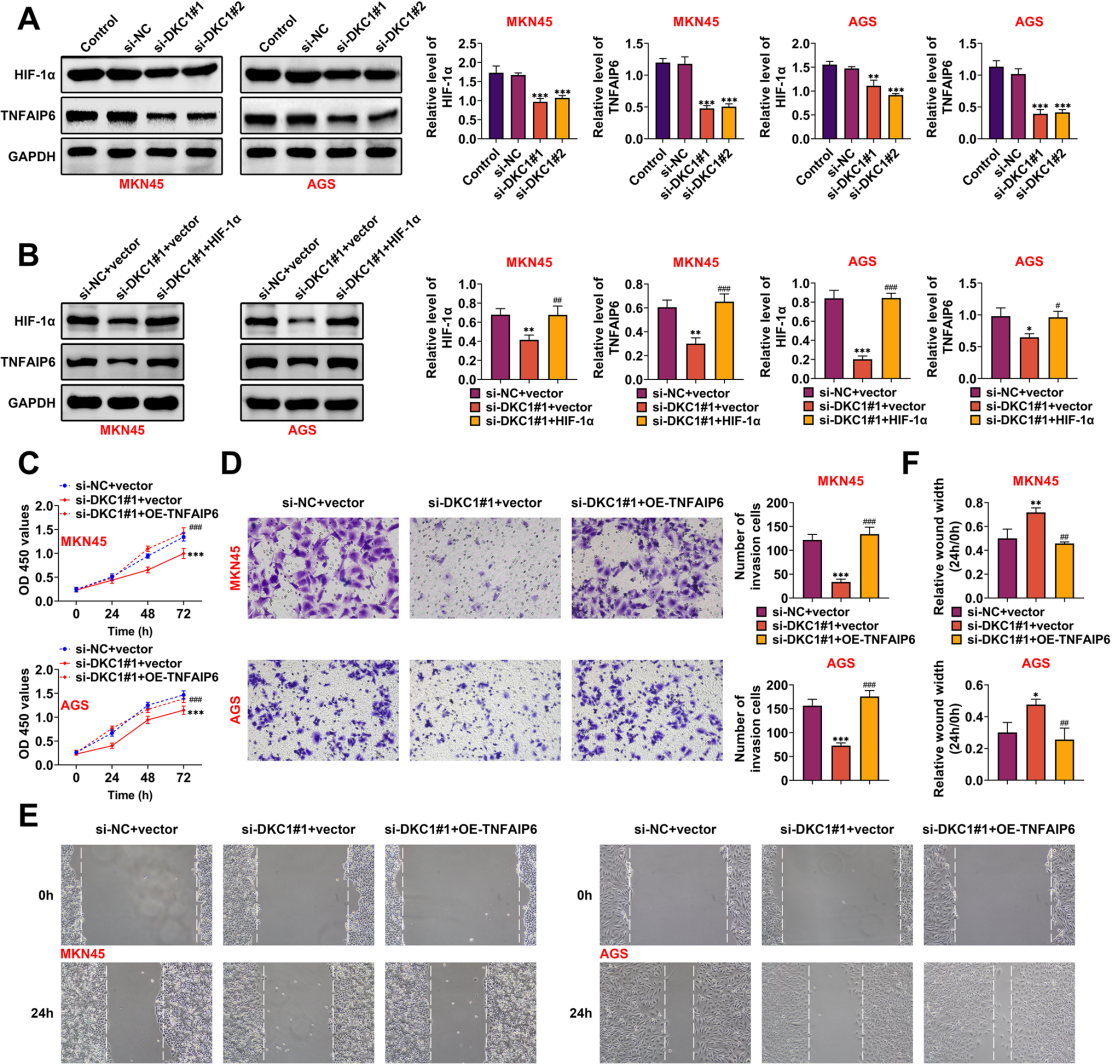
DKC1 modulated TNFAIP6 to aggravate GC progression. **A** The protein expressions of HIF-1α and TNFAIP6 were examined after DKC1 suppression in MKN45 and AGS cells through western blot. **B** The protein expressions of HIF-1α and TNFAIP6 were tested through western blot. Groups were divided into the si-NC + vector, si-DKC1#1 + vector, and si-DKC1#1 + HIF-1α group. **C** The cell proliferation was detected in MKN45 and AGS cells through CCK-8 assay. Groups were divided into the si-NC + vector, si-DKC1#1 + vector, and si-DKC1#1 + OE-TNFAIP6 group. **D** The cell invasion was measured in MKN45 and AGS cells through Transwell assay. Groups were divided into the si-NC + vector, si-DKC1#1 + vector, and si-DKC1#1 + OE-TNFAIP6 group. **E**, **F** The cell migration was examined in MKN45 and AGS cells through wound healing assay. Groups were divided into the si-NC + vector, si-DKC1#1 + vector, and si-DKC1#1 + OE-TNFAIP6 group. *N* = 3. *p < 0.05, **p < 0.01, ***p < 0.001 vs the si-NC + vector group; ##*p* < 0.01, ###*p* < 0.001 vs the si-DKC1#1 + vector group

TNFAIP6 expression has been discovered to be elevated in HIF-1α-induced lung cancer cells (Wan et al. 2011). However, the relationship between DKC1 and TNFAIP6 in GC progression needs further studies. The protein expressions of HIF-1α and TNFAIP6 were both lessened after DKC1 knockdown (Fig. 4A) (*p* < 0.01). The decreased protein expressions of HIF-1α and TNFAIP6 mediated by DKC1 knockdown can be reversed after HIF-1α overexpression (Fig. 4B). Next, rescue assays were done to verify the regulatory relationship between DKC1 and TNFAIP6 in GC. The decreased cell proliferation mediated by DKC1 inhibition could be reversed by TNFAIP6 overexpression (Fig. 4C) (*p* < 0.001). Besides, the cell invasion was reduced after DKC1 repression, but this change was offset by TNFAIP6 upregulation (Fig. 4D) (*p* < 0.001). Furthermore, the weakened cell migration ability mediated by suppressing DKC1 was rescued by overexpressing TNFAIP6 (Fig. 4E, F) (*p* < 0.05). In summary, DKC1 modulated TNFAIP6 to aggravate GC progression.

### DKC1 strengthened tumor growth in vivo

**Fig. 5:**
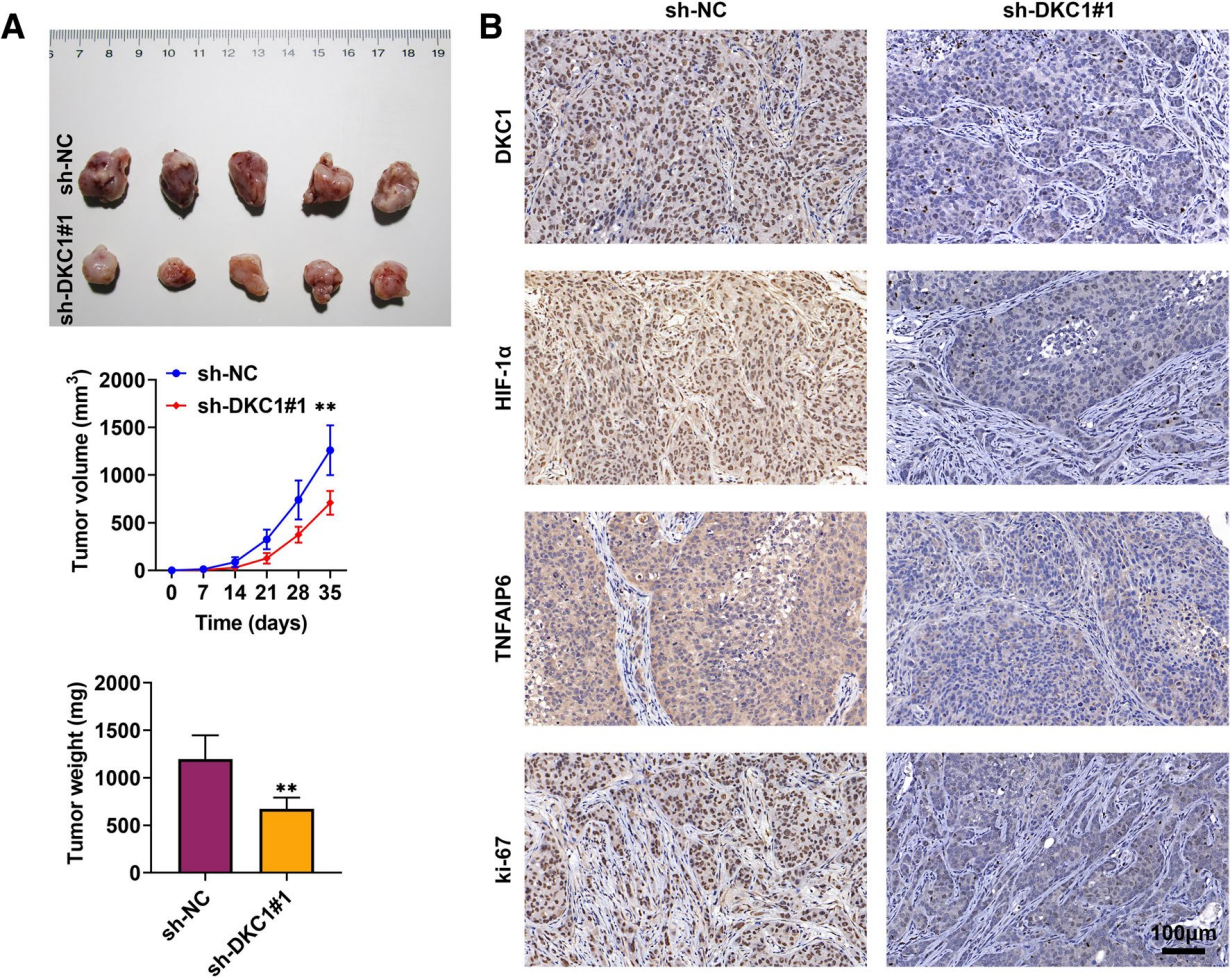
DKC1 strengthened tumor growth in vivo. **A** The tumor size, volume, and weight were assessed after silencing DKC1 through in vivo assay. **B** The protein expressions of DKC1, HIF-1α, TNFAIP6, and ki-67 were measured after inhibiting DKC1 through IHC assay. *N* = 5. **p < 0.01

The in vivo experiments were carried out to deeply probe the effect of DKC1 in GC progression. The size, volume, and weight of tumors were decreased after DKC1 suppression (Fig. 5A) (*p* < 0.01). Additionally, through IHC assay, the protein expressions of DKC1, HIF-1α, TNFAIP6, and ki-67 were downregulated after DKC1 inhibition (Fig. 5B). These data suggested that DKC1 strengthened tumor growth in vivo.

## Discussion

Plenty of proteins have been demonstrated to participate into GC progression. For example, RNA-binding protein RNPC1 stabilizes aurora kinase B mRNA to exacerbate GC progression (Ji et al. 2021). Furthermore, placenta-specific protein 1 modulates the AKT/GSK-3β/cyclin D1 signaling pathway to strengthen tumorigenesis in GC (Liu et al. 2021). Additionally, CCDC65 enhances ENO1 ubiquitination to suppress the AKT1 activation in GC (Deng et al. 2021). Jab1 regulates the non-ubiquitin proteasomal degradation of p14ARF to heighten GC tumorigenesis (Wang et al. 2020). Thus, seeking novel and effective proteins is crucial for GC therapeutic strategies.

DKC1 has been affirmed to be a serviceable facilitator in diversiform cancers (Hou et al. 2020; Kan et al. 2021; Miao et al. 2019; Zhang et al. 2018; Yang et al. 2020; Kim et al. 2012), but its function in GC maintains dimness. In this study, at first, DKC1 expression was discovered to be upregulated in GC tissues through GEPIA, UALCAN, and TIMER databases. Moreover, it was identified that DKC1 exhibited higher expression in GC cells. Functional experiments testified that DKC1 accelerated cell proliferation, migration, and invasion in GC.

TNFα-stimulated gene-6 (TNFAIP6), also known as TSG6, is a member of the hyaluronic acid–binding protein family and exhibits a pivotal role in the protease network associated with inflammation (Evrard et al. 2021; Yu et al. 2016; Rajer et al. 2021). TNFAIP6 has been also joined in extracellular matrix (ECM) remodeling, cell adhesion, and migration to regulate cancer progression. For example, higher expression of TNFAIP6 results into poor prognosis in urothelial carcinomas (Chan et al. 2019). Besides, TNFAIP6 facilitates aggressiveness in a CD44-dependent manner to enhance metastasis in colorectal cancer (Liu et al. 2022). What is more, the higher expression of TNFAIP6 in GC tissues was discovered than that in normal gastric tissues, and its level was positively relevant with lymph node metastasis and TNM stage. And, TNFAIP6 knockdown in GC restrains cell proliferation, invasion, and metastasis (Zhang et al. 2021).

Researchers have discovered that the mRNA and protein expressions of HIF-1α are undetectable in most normal tissues, but overexpressed in a variety of human cancers, and HIF-1α exacerbates tumorigenesis, metastasis, and angiogenesis (Lin et al. 2017; Semenza 2003; Masoud and Li 2015). It is uncovered that TNFAIP6 expression is elevated in HIF-1α-induced lung cancer cells (Wan et al. 2011). However, the relationship between DKC1 and TNFAIP6 in GC progression needs further studies. In this work, it was demonstrated that the protein levels of HIF-1α and TNFAIP6 were decreased after silencing DKC1. Further investigation indicated that DKC1 modulated TNFAIP6 to aggravate GC progression; the decreased cell proliferation, migration, and invasion stimulated by DKC1 knockdown could be rescued after TNFAIP6 overexpression. Lastly, through in vivo experiments, it was demonstrated that DKC1 strengthened tumor growth.

To sum up, it was the first time to illuminate that DKC1 aggravated GC cell migration and invasion by upregulating the expression of TNFAIP6. However, our current work has some limitations for the influences of DKC1 on GC progression. In the future, more experiments were made to further investigate the other regulatory roles of DKC1 in GC.

## Data Availability

All data generated or analyzed during this study are included in this published article. The datasets used and/or analyzed during the present study are available from the corresponding author on reasonable request.
